# Endogenous Bioelectrical Modulation by REAC Metabolic Optimization-IBZ Modulates SIRT1, PPAR-γ, and Metabolic Signaling Pathways in Human Fibroblasts

**DOI:** 10.3390/cells15020106

**Published:** 2026-01-07

**Authors:** Sara Cruciani, Vania Fontani, Arianna Rinaldi, Salvatore Rinaldi, Margherita Maioli

**Affiliations:** 1 Department of Biomedical Sciences, University of Sassari, 07100 Sassari, Italy; sara.cruciani@outlook.com (S.C.); mmaioli@uniss.it (M.M.); 2 Department of Reparative and Regenerative Medicine, Rinaldi Fontani Institute, 50144 Florence, Italy; vfontani@irf.it (V.F.); ari@irf.it (A.R.); 3 Research Department, Rinaldi Fontani Foundation, 50144 Florence, Italy

**Keywords:** human fibroblasts, endogenous bioelectrical activity, bioelectrical modulation, metabolic homeostasis, SIRT1, PPAR-γ, epigenetic modulation, cellular reprogramming, regenerative medicine, non-invasive treatment

## Abstract

**Highlights:**

**What are the main findings?**

- REAC Metabolic Optimization-IBZ induces a coordinated downregulation of SIRT1 and upregulation of PPAR-γ in human fibroblasts, representing a molecular profile plausibly associated with changes in cellular metabolic regulation, although functional metabolic consequences were not directly evaluated in this study.
- REAC MO-IBZ is associated with directional increases in key metabolic and energetic pathway proteins in fibroblasts, including mTOR, IGF-1R, and cytochrome c, based on qualitative immunofluorescence evidence, supporting further investigation of REAC-based bioelectrical modulation and its impact on cellular bioenergetic regulation.

**What are the implications of the main findings?**

- The observed gene and protein expression profile suggests that REAC MO-IBZ may promote a biologically coherent modulation of regulatory pathways, potentially contributing to functional cellular reprogramming through endogenous bioelectrical and epigenetic mechanisms. However, these mechanisms were not directly investigated and should be interpreted as hypothesis-generating.
- These findings provide preliminary mechanistic indications supporting the use of REAC-based bioelectrical modulation as a non-invasive strategy in regenerative medicine and metabolic dysfunction-related conditions, while recognizing that additional functional and quantitative analyses are required to substantiate direct physiological effects.

**Abstract:**

Fibroblasts play a fundamental role in maintaining tissue architecture, regulating repair processes, and adapting to metabolic and inflammatory stress. Increasing evidence indicates that endogenous bioelectrical states contribute to gene expression regulation and cellular homeostasis. In this study, we investigated the effects of Radio Electric Asymmetric Conveyer (REAC) Metabolic Optimization–Inside Blue Zone (MO-IBZ) treatment on key regulators of stress response and metabolic control in human foreskin fibroblasts (HFF-1). Cells were exposed to nine standardized REAC MO-IBZ sessions, and changes in gene and protein expression were evaluated. Quantitative RT-PCR revealed a significant downregulation of SIRT1 and an upregulation of PPAR-γ expression in treated cells compared with untreated controls. These findings indicate molecular changes involving stress-responsive and metabolic regulatory pathways; however, they should be interpreted primarily as transcriptional signatures, as no direct functional stress-response or metabolic assays were performed. Immunofluorescence analysis showed visually increased expression of mTOR, IGF-1 receptor, and cytochrome c in REAC-treated fibroblasts, supporting a qualitative indication of activation of pathways associated with anabolic signaling, mitochondrial function, and metabolic efficiency. Taken together, these findings indicate that REAC MO-IBZ induces a coordinated molecular profile compatible with changes in cellular metabolic regulatory capacity. Within the framework of current bioelectrical literature, these changes may plausibly reflect broader regulatory adaptations; however, the present work does not provide direct measurements of bioelectrical parameters, functional metabolic activity, or epigenetic regulation, and therefore such interpretations remain speculative. These results provide descriptive mechanistic evidence supporting further investigation of REAC-based bioelectrical modulation as a potential strategy to influence cellular pathways involved in metabolic balance and tissue repair, encouraging future studies incorporating direct bioelectrical, epigenetic, and functional analyses.

## 1. Introduction

Cellular responses to chronic stress [1], inflammation [2], and metabolic imbalance [3] are mediated not only by biochemical signaling but also by alterations in endogenous bioelectrical activity [4,5], which are increasingly recognized as modulators of gene expression through epigenetic mechanisms [1].

HFF-1 fibroblasts represent a widely used and reproducible in vitro model to investigate metabolic, stress-related, and reparative regulatory mechanisms in human connective tissue cells [6,7,8]. These cells are not passive structural components but active participants in sensing and responding to environmental cues.

Importantly, fibroblasts exhibit substantial epigenetic plasticity, enabling them to integrate environmental, metabolic, and mechanical signals into stable transcriptional programs that govern tissue homeostasis and repair [9]. Despite their central role in coordinating metabolic adaptation, tissue repair, and inflammatory responses, the integrated regulation of SIRT1 and PPAR-γ in these cells remains poorly characterized. Increasing evidence indicates that fibroblasts integrate metabolic, inflammatory, and bioenergetic cues to recalibrate their transcriptional and functional state, particularly under adverse or prolonged stress conditions.

In this context, pathways involving SIRT1, PPAR-γ, IGF-1R, and mTOR signaling have emerged as central hubs in coordinating cellular adaptation, influencing mitochondrial dynamics, oxidative balance, reparative potential, and metabolic efficiency. Understanding how these regulatory axes may be modulated upstream through non-pharmacological approaches capable of engaging endogenous regulatory mechanisms therefore represents a relevant scientific objective. Within this framework, the investigation of molecular signatures induced by controlled bioelectrical modulation may contribute to advancing current knowledge on cellular regulatory plasticity and stress-related adaptation in human fibroblasts. Although endogenous bioelectrical signaling is increasingly recognized as a regulator of chromatin dynamics and gene expression, no studies to date have investigated how bioelectrical modulation may influence these key metabolic and stress-responsive genes in human fibroblasts.

Among the molecular regulators governing the fibroblast response to environmental and metabolic stress, Sirtuin 1 (SIRT1) [10] and peroxisome proliferator-activated receptor gamma (PPAR-γ) [11] are particularly significant. SIRT1, a NAD+-dependent deacetylase, is involved in DNA repair, oxidative stress response, and metabolic regulation [11]. It is frequently upregulated in pathological states as a compensatory response to cellular distress [10]. In contrast, PPAR-γ is a nuclear receptor known to promote lipid storage [12], improve insulin sensitivity [13], and exert anti-inflammatory effects [14].

In the context of fibroblasts, PPAR-γ contributes to maintaining tissue homeostasis and regulating differentiation [15].

Understanding how SIRT1 and PPAR-γ are regulated in fibroblasts is particularly relevant because these cells orchestrate extracellular matrix remodeling, metabolic balance, and inflammatory resolution, making them a strategic target for interventions aimed at restoring tissue integrity under stress conditions.

Mitochondria are central to cellular metabolism [16]. In this context, Cytochrome C (Cyt C) is an electron carrier in the mitochondrial respiratory chain that acts as both a metabolic regulator and a mediator of programmed cell death in fibroblasts, helping maintain tissue homeostasis [17]. It is also involved in the early phases of apoptosis and is released following a strong oxidative stress event [17]. The mammalian target of rapamycin (mTOR) and insulin-like growth factor-1 receptor (IGF-1R) pathways also exert a pivotal role in regulating cell growth and energy metabolism [17,18]. Cellular starvation occurs when cells are deprived of nutrients or oxygen, triggering adaptive mechanisms to survive until nutrients become available again [19]. Under starvation, cells modulate fatty acid trafficking and hypoxic growth responses through the modulation of mTOR. mTOR is co-regulated by IGF-1R, promotes de novo synthesis of glycine, the most abundant amino acid in collagen, inducing its production by fibroblasts in response to environmental stress.

Bioelectricity is a multifaceted signal essential in regulating the functioning of both individual cells and more complex biological processes [20,21]. The cellular bioelectric state is an overall signal derived from multiple ion channels, pumps, gap junctions that finely regulate proliferation, differentiation, metabolism and cell migration [21,22]. Recent advances revealed that alterations in bioelectrical gradients can influence chromatin structure, histone modification, and DNA methylation status, the hallmarks of epigenetic regulation [22,23].

From a biological perspective, bioelectrical mechanisms provide a plausible explanatory framework for these observations; however, the present study was not designed to directly demonstrate such mechanisms or related epigenetic modifications, and these aspects must therefore be considered speculative.

Radio Electric Asymmetric Conveyer (REAC) technology harnesses low-intensity, asymmetrically conveyed radio electric fields to restore physiological bioelectrical states that are often disrupted in chronic pathological conditions [24].

These fields do not directly alter DNA sequences; nevertheless, they can influence ion fluxes, membrane potentials, and intracellular signaling cascades, which may in turn modulate the activity of enzymes involved in epigenetic regulation [22,23].

However, the current study does not experimentally address these mechanistic steps and focuses instead on measurable molecular endpoints.

The Metabolic Optimization–Inside Blue Zone (MO-IBZ) protocol of REAC is designed to modulate the cellular response to metabolic dysfunction by acting upstream of classical signaling pathways, at the level of the cell’s intrinsic bioelectrical framework.

In this study, we examine the effects of REAC MO-IBZ on the expression of SIRT1 and PPAR-γ in HFF-1 fibroblasts and interpret these findings within a framework that considers potential bioelectrical and epigenetic regulatory interactions that could underlie the beneficial clinical effects of REAC treatments.

## 2. Materials and Methods

### 2.1. Cell Culture and REAC MO-IBZ Treatment

Human foreskin fibroblasts (HFF-1), obtained from ATCC (Manassas, VA, USA), were cultured in Dulbecco’s Modified Eagle’s Medium (DMEM; Life Technologies, Carlsbad, CA, USA) supplemented with 10% fetal bovine serum (FBS; Life Technologies), 2 mM L-glutamine (Euroclone, Milan, Italy), and 1% penicillin/streptomycin (Euroclone, Milan, Italy). Cells were grown to 70–80% confluence prior to treatment.

The REAC MO-IBZ protocol was administered using the BENE 110 device (ASMED, Scandicci, Italy), specifically configured for Inside Blue Zone metabolic optimization. Treatment consisted of nine sessions of 30 min each, administered on consecutive days. Asymmetric Conveyor Probes (ACPs) were applied in direct contact with the culture flask, acting as asymmetric conveyors that channel the interaction of the radio-electric field generated by the REAC device toward the cultured cells.

Untreated control cultures were maintained under identical environmental, handling, and incubation conditions, differing only in the absence of REAC MO-IBZ application. The number, duration, and timing of REAC MO-IBZ sessions were defined by preset protocol parameters that cannot be modified by the operator, ensuring standardization, reproducibility, and consistency across experiments [25]. To minimize procedural bias, cultures were processed in parallel and handled by the same operators under identical laboratory conditions. Particular care was taken to minimize environmental variability during all procedures. Cell culture handling, incubation times, and treatment exposure were standardized across independent experiments, and cell passages were kept within a controlled range to avoid phenotypic drift. The allocation of culture plates to experimental conditions was managed to reduce procedural bias, and replicate samples were processed in parallel whenever possible. These precautions were implemented to ensure the stability of culture conditions and improve the robustness of the molecular observations obtained.

### 2.2. Gene Expression Analysis

Following treatment, total RNA was isolated from control and REAC MO-IBZ-treated HFF-1 cells using the RNeasy Mini Kit (Qiagen, Hilden, Germany). RNA quality and quantity were assessed spectrophotometrically. Real-time quantitative PCR was performed using the Luna^®^ Universal One-Step RT-qPCR Kit (New England Biolabs, Ipswich, MA, USA) on a CFX Thermal Cycler (Bio-Rad, Hercules, CA, USA), with specific primers for SIRT1 and PPAR-γ. GAPDH was used as the reference gene. Relative expression levels were calculated using the 2^−ΔΔCt^ method. Experiments were performed in three independent biological replicates, each including technical triplicates.

Statistical comparisons were based on biological replicates, while technical replicates were used only to ensure analytical reliability.

### 2.3. High-Resolution Fluorescence Microscopy with Computational Clearing

High-resolution fluorescence microscopy with computational clearing was used to assess the expression of SIRT1, mTOR, cytochrome c, and IGF-1R in HFF-1 cells following REAC MO-IBZ treatment. Cells were fixed in 10% neutral buffered formalin (approximately 4% formaldehyde) (Sigma Aldrich Chemie GmbH, 82024 Taufkirchen, Germany) and permeabilized with 0.1% Triton X-100 in PBS (Thermo Fisher Scientific, Grand Island, NY, USA). Primary antibodies against SIRT1 (Cell Signaling, Danvers, MA, USA), mTOR (Abcam, Cambridge, UK), cytochrome c (Cell Signaling, Danvers, MA, USA), and IGF-1R (Cell Signaling, Danvers, MA, USA) were incubated overnight at 4 °C under gentle agitation. Cells were then washed in PBS and incubated with fluorescence-conjugated secondary antibodies (Life Technologies, USA) for 1 h at 37 °C in the dark. Nuclei were labeled with DAPI (1 μg/mL) (Thermo Fisher Scientific, Grand Island, NY, USA). Fluorescence images were acquired using a THUNDER Imager DMi8 microscope (Leica, Nussloch, Germany). All analyses were performed in three independent biological experiments.

Image acquisition parameters, including exposure time, gain, illumination intensity, and resolution, were maintained constant throughout experiments to favor comparability between samples. Images were collected from multiple fields within each sample, avoiding oversaturated areas and ensuring adequate signal distribution. Representative fields were selected on the basis of technical quality and morphological suitability rather than signal intensity alone, in order to provide a realistic visualization of the qualitative molecular patterns observed.

Protein expression analysis was qualitative in nature; therefore, fluorescence microscopy findings are presented as representative images rather than quantitative measurements.

### 2.4. Statistical Analysis

Statistical analysis was performed using GraphPad Prism 9.0 software (GraphPad, San Diego, CA, USA). Since data were not normally distributed, comparisons between control and REAC MO-IBZ-treated cells were performed using the Mann–Whitney U test. A *p* value < 0.05 was considered statistically significant. Statistical significance was reported as *p* < 0.05, *p* < 0.01, *p* < 0.001, and *p* ≤ 0.0001.

Statistical analysis applies exclusively to gene expression datasets, whereas immunofluorescence data are presented descriptively. Statistical analyses and data handling were conducted with particular attention to transparency and reproducibility. The choice of statistical approaches was based on the distribution characteristics of the available data and on the descriptive mechanistic nature of the study. As the primary aim of this work was the characterization of molecular regulatory tendencies rather than the measurement of quantitative functional outcomes, statistical interpretation was intentionally conservative and aligned with the exploratory scope of the investigation.

## 3. Results

### 3.1. REAC-MO Treatment Resulted in Significant Transcriptional Modulation of Key Genes Associated with Cellular Stress and Metabolic Regulation

**Figure 1:**
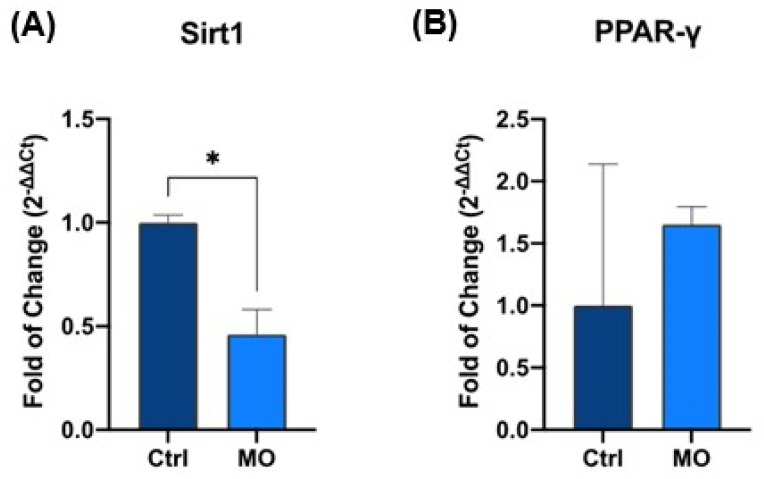
Expression of SIRT1 (**A**) and PPAR-γ (**B**) in control vs. REAC MO-IBZ-treated HFF-1. The mRNA levels for each gene were normalized to Glyceraldehyde-3-Phosphate-Dehydrogenase (hGAPDH) and expressed as fold of change (2^−∆∆Ct^) of the mRNA levels observed in untreated controls defined as 1. Data are expressed as mean ± SD (* *p* ≤ 0.05).

Quantitative real-time PCR analysis revealed that SIRT1 expression was significantly decreased in fibroblasts treated with REAC MO-IBZ compared to untreated controls (*p* < 0.05) (Figure 1A).

This reduction is consistent with modulation of stress-responsive regulatory pathways; however, its precise functional meaning should be interpreted cautiously in the absence of direct functional assays [26,27,28,29].

Conversely, PPARγ expression was upregulated in the treated group (Figure 1B), supporting the involvement of transcriptional programs related to metabolic regulation and anti-inflammatory signaling. The dual and opposing modulation of these genes (Figure 1A,B) is consistent with a coordinated regulatory shift, plausibly associated with changes in stress-related and metabolic control pathways, although this interpretation remains inferential in the absence of direct functional analyses [30,31].

Together, these results provide molecular evidence that REAC MO-IBZ treatment modulates stress- and metabolism-associated gene expression, representing biologically meaningful transcriptional signatures that warrant further functional investigation [31].

### 3.2. REAC MO-IBZ Treatment Is Associated with Qualitative Modulation of Metabolic Regulatory Proteins

**Figure 2:**
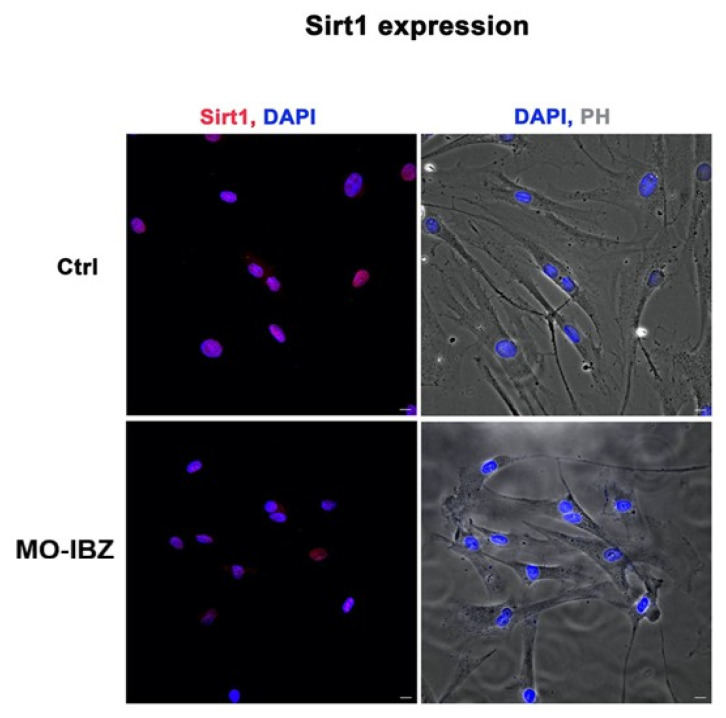
Fluorescence microscopy with computational clearing showing SIRT1 expression in HFF-1 cells after REAC MO-IBZ treatment compared with untreated controls. Nuclei are labeled with 4′,6-diamidino-2-phenylindole (DAPI; blue). Scale bar: 40 μm. Images are representative of independent biological experiments.

Fluorescence microscopy with computational clearing analysis confirmed that SIRT1 expression was significantly decreased in fibroblasts treated with REAC MO-IBZ [26,27,28,29] compared to untreated controls (Figure 2).

These observations are consistent with PCR findings but should be considered descriptive rather than quantitatively conclusive.

**Figure 3:**
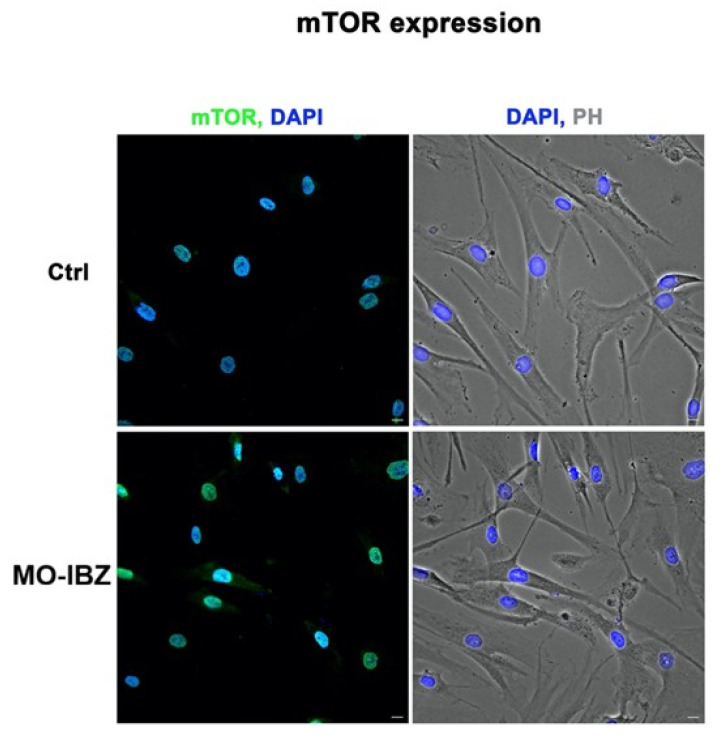
Fluorescence microscopy with computational clearing showing mTOR expression in HFF-1 cells after REAC MO-IBZ treatment compared with untreated controls. Nuclei are labeled with 4′,6-diamidino-2-phenylindole (DAPI; blue). Scale bar: 40 μm. Images are representative of independent biological experiments.

**Figure 4:**
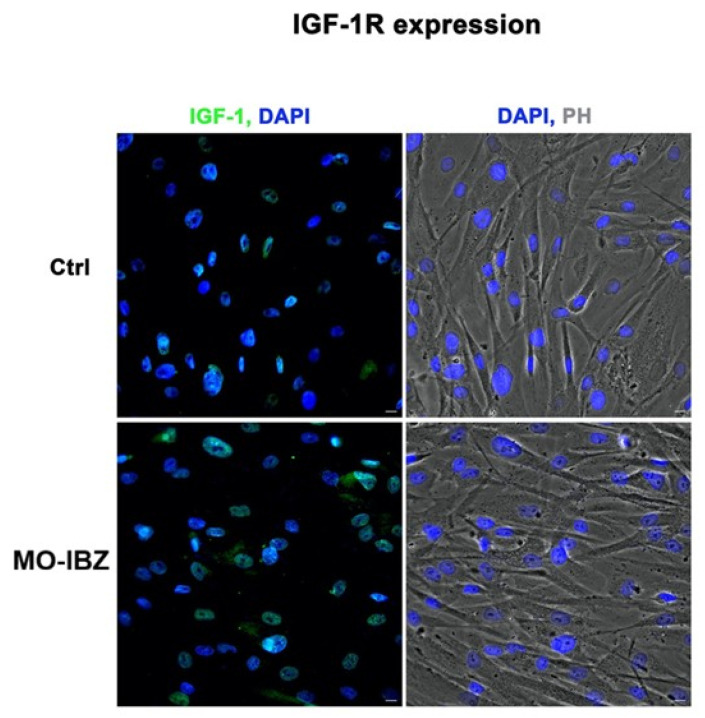
Fluorescence microscopy with computational clearing showing IGF-1R expression in HFF-1 cells after REAC MO-IBZ treatment compared with untreated controls. Nuclei are labeled with 4′,6-diamidino-2-phenylindole (DAPI; blue). Scale bar: 40 μm. Images are representative of independent biological experiments.

**Figure 5:**
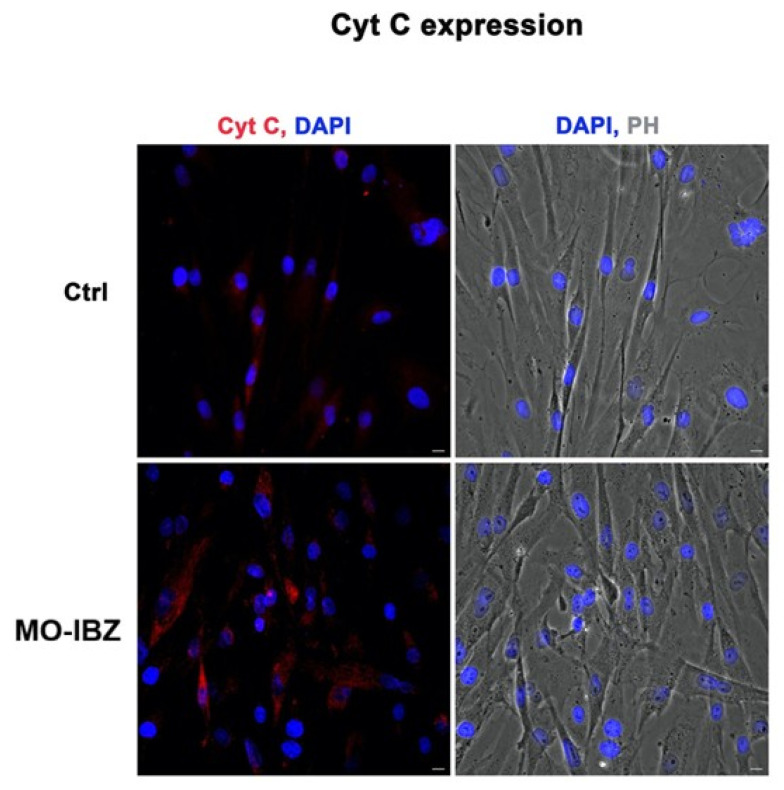
Fluorescence microscopy with computational clearing showing cytochrome c expression in HFF-1 cells after REAC MO-IBZ treatment compared with untreated controls. Nuclei are labeled with 4′,6-diamidino-2-phenylindole (DAPI; blue). Scale bar: 40 μm. Images are representative of independent biological experiments.

Simultaneously, we observed a marked increase in the expression of mTOR [32,33] (Figure 3), IGF-1R 42 (Figure 4), and Cytochrome c [34,35] (Figure 5) in REAC MO-IBZ–treated HFF-1 cells compared to untreated controls, This pattern supports the qualitative indication that REAC MO-IBZ may influence pathways associated with metabolic regulation, cellular energy balance, and mitochondrial activity.

Given the qualitative nature of immunofluorescence, these observations indicate directional trends rather than demonstrating statistically quantified protein increases. Future quantitative analyses (e.g., Western blotting or image-based fluorescence quantification) will be required to confirm the magnitude of these effects.

## 4. Discussion

The results of this study demonstrate that REAC MO-IBZ treatment induces coordinated and biologically meaningful transcriptional and protein-level changes in human fibroblasts, consistent with the modulation of pathways involved in metabolic regulation and cellular homeostasis. The observed molecular profile suggests engagement of broader regulatory networks governing cellular adaptation. These findings should be interpreted primarily as descriptive molecular signatures that indicate biologically coherent modulation of regulatory pathways, rather than as definitive evidence of functional metabolic enhancement or stable cellular reprogramming, since no direct functional metabolic or stress-response assays were included in the present study [22,23,36,37,38].

A central finding of this work is the significant reduction in SIRT1 expression following REAC MO-IBZ treatment. Although SIRT1 is widely recognized as a factor involved in cellular protection, longevity-associated pathways, and stress resilience, its upregulation is also commonly interpreted as a compensatory response to oxidative or metabolic stress.

In this framework, the downregulation of SIRT1 observed in REAC-treated fibroblasts may plausibly reflect a reduced requirement for stress-compensatory mechanisms [1,10]. At the same time, we acknowledge that SIRT1 biology is context-dependent, and therefore alternative interpretations cannot be excluded, including the possibility that reduced expression may in some settings correspond to altered or redistributed stress-regulatory demands rather than uniformly improved balance. Future studies integrating direct functional, metabolic, and electrophysiological assessments will be essential to determine whether these molecular signatures translate into measurable biological effects.

This interpretation aligns with evidence showing that SIRT1 expression increases under conditions of metabolic imbalance and oxidative burden, functioning as a stress-sensing regulator whose downregulation is compatible with restored homeostatic conditions [39].

This cautious interpretation is further supported by the concomitant upregulation of regulatory pathways classically associated with metabolic efficiency and energy control. The increased expression of PPAR-γ suggests engagement of transcriptional programs linked to lipid handling, glucose regulation, and anti-inflammatory mechanisms [11,14].

Beyond its metabolic role, PPAR-γ is also known to inhibit pro-fibrotic signaling and promote controlled differentiation, processes particularly relevant in fibroblast biology and tissue remodeling [31]. Taken together, the coordinated modulation of SIRT1 and PPAR-γ delineates a biologically coherent molecular pattern suggestive of regulatory adjustment rather than random fluctuation; however, such coherence should be considered mechanistic indication rather than proof of functional reorganization.

The fluorescence microscopy findings provide additional support for this interpretation. REAC MO-IBZ treatment was associated with enhanced immunofluorescence patterns of mTOR, IGF-1R, and cytochrome c, molecules known to be central to energy sensing, anabolic signaling, and mitochondrial function [32,33,34,35,40].

These data indicate directional trends toward increased metabolic pathway engagement; nevertheless, as highlighted by both reviewers, the qualitative nature of immunofluorescence requires caution. For this reason, we have intentionally avoided referring to these changes as “significant increases” and instead present them as visually appreciable modifications consistent with the observed transcriptional profile. Future studies incorporating Western blotting, fluorescence quantification, or flow cytometry will be essential to validate these patterns quantitatively.

The interplay between SIRT1- and mTOR-related pathways deserves particular attention. These systems form a tightly interconnected regulatory axis that integrates nutrient availability, mitochondrial performance, and stress-response programs [41,42,43]. The simultaneous directional modulation of these systems in our study strengthens the biological plausibility that REAC MO-IBZ may influence upstream regulatory hubs. However, mechanistic causality cannot be inferred in the absence of direct bioelectrical measurements, metabolic flux analyses, or targeted pathway perturbation experiments.

An additional conceptual dimension emerging from this study relates to bioelectrical regulation. Increasing evidence indicates that endogenous bioelectrical states contribute to controlling transcriptional architecture and cellular identity by influencing ion fluxes, membrane potential, chromatin accessibility, and enzyme activity [21,22,23]. Within this framework, REAC MO-IBZ has been hypothesized to act by interacting with endogenous bioelectrical dynamics [24,44]. In this light, the molecular profile observed here is best understood as a coherent biological response compatible with modulation of upstream regulatory systems, without implying direct demonstration of mechanistic causality or functional benefit.

Finally, although this in vitro investigation does not aim to establish clinical efficacy, it contributes mechanistic insight that may assist in understanding previously reported biological and translational outcomes involving REAC-based treatments [44,45,46,47]. This connection is discussed with appropriate caution, particularly in light of potential conflicts of interest. For this reason, we have deliberately maintained a conservative interpretative approach, limiting conclusions to objectively measurable biological endpoints while indicating translational implications only as perspectives requiring further confirmation.

Overall, the present findings support the concept that REAC MO-IBZ represents a non-invasive strategy capable of modulating molecular pathways associated with metabolic regulation, mitochondrial function, and stress-response signaling in fibroblasts. While the observations presented here establish reproducible and biologically coherent molecular changes, future work will need to consolidate these findings by quantifying protein-level modifications, integrating functional metabolic analyses, and extending evaluation beyond a single fibroblast cell line. From a broader perspective, the present findings provide preliminary but biologically coherent molecular insights into how upstream modulation strategies may interact with cellular regulatory architecture in human fibroblasts. Although the study was not designed to evaluate functional performance, bioenergetic efficiency, or electrophysiological dynamics, the present evidence contributes to refining the conceptual framework through which bioelectrical modulation is considered within cellular stress adaptation research. Future studies including quantitative protein evaluation, direct metabolic assessment, and integrative epigenetic or electrophysiological analyses will be essential to confirm and expand the significance of these observations, further clarifying their potential translational relevance.

## 5. Conclusions

The results of this study demonstrate that REAC MO-IBZ treatment induces coordinated and reproducible molecular changes in human fibroblasts, characterized by downregulation of SIRT1 and upregulation of PPAR-γ at the transcriptional level, together with qualitative enhancement of mTOR, IGF-1R, and cytochrome c expression. This integrated molecular profile is biologically coherent with regulatory adaptations involving metabolic control and cellular stress-response pathways, although functional consequences cannot be inferred directly from the present dataset.

These observations should therefore be interpreted as mechanistic evidence at the molecular level rather than as proof of functional enhancement, and they encourage further investigation into the cellular processes potentially influenced by REAC-induced bioelectrical modulation. In particular, future studies incorporating functional metabolic assays, bioelectrical measurements, epigenetic profiling, and evaluation across multiple cell models will be fundamental to defining the specificity, durability, and physiological relevance of these findings.

Within this scientifically cautious framework, the present study contributes a significant mechanistic step forward by providing structured and biologically meaningful molecular evidence supporting the hypothesis that REAC MO-IBZ may interact with upstream regulatory networks involved in metabolic stability and tissue maintenance. These insights strengthen the rationale for continued exploration of bioelectrical modulation strategies as potential tools to support cellular homeostasis and regenerative processes. In summary, this study provides descriptive molecular evidence indicating that controlled endogenous bioelectrical modulation is associated with coherent regulatory adjustments in key molecular pathways linked to cellular homeostasis and metabolic control in human fibroblasts. While these results must be interpreted within the methodological limits of the present study, they support the rationale for further investigation into upstream modulation strategies as potential tools to better understand cellular regulatory plasticity. Future research integrating molecular, functional, and quantitative analyses will be decisive in determining the broader biological implications of these findings.

## Data Availability

All data supporting the findings of this study are contained within the manuscript.
